# Author Correction: Rapid cost decrease of renewables and storage accelerates the decarbonization of China’s power system

**DOI:** 10.1038/s41467-020-17706-3

**Published:** 2020-07-24

**Authors:** Gang He, Jiang Lin, Froylan Sifuentes, Xu Liu, Nikit Abhyankar, Amol Phadke

**Affiliations:** 1 Department of Technology and Society, College of Engineering and Applied Sciences, Stony Brook University, Stony Brook, NY 11794 USA; 2 International Energy Analysis Department, Lawrence Berkeley National Laboratory, Berkeley, CA 94720 USA; 3 Department of Agricultural and Resources Economics, University of California, Berkeley, Berkeley, CA 94720 USA; 4 Institute of Energy Studies, Environmental Sciences Department, Western Washington University, Bellingham, WA 98225 USA

**Keywords:** Climate-change mitigation, Energy and society

Correction to: *Nature Communications* 10.1038/s41467-020-16184-x, published online 19 May 2020.

The current Figs. 1 and 2 have incorrect numbers shown on the purple bars. The source data was also incorrect in the original version of the Article.

This has been corrected in both the PDF and HTML versions of the Article.

