# Supplementary material for: Rapid cost decrease of renewables and storage accelerates the decarbonization of China’s power system
Source: Nat Commun. 2020 May 19;11:2486. doi: 10.1038/s41467-020-16184-x (PMC7237428; doi:10.1038/s41467-020-16184-x)
Supplement: Supplementary file 1 — Supplementary Information [file 41467_2020_16184_MOESM1_ESM.pdf]

Supplementary Information

Rapid cost decrease of renewables and storage accelerates the decarbonization of China's power system

He et al.

This file includes:

Supplementary Figures 1, 2.

Supplementary Tables 1.

Supplementary Notes 1, 2, 3, 4, 5.

Supplementary References.

Supplementary Figures

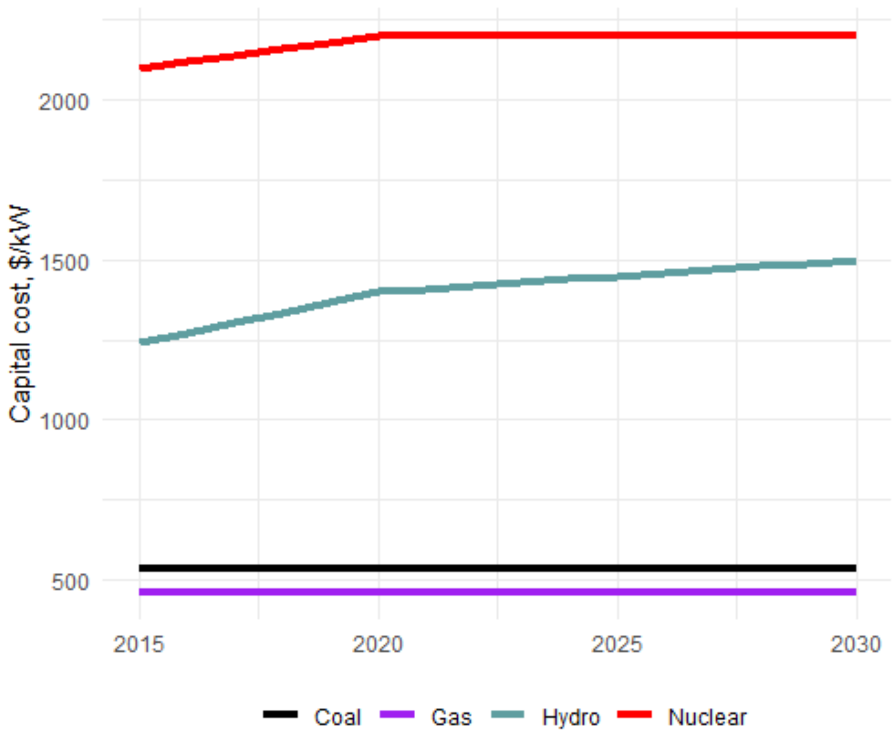

(a) Capital costs

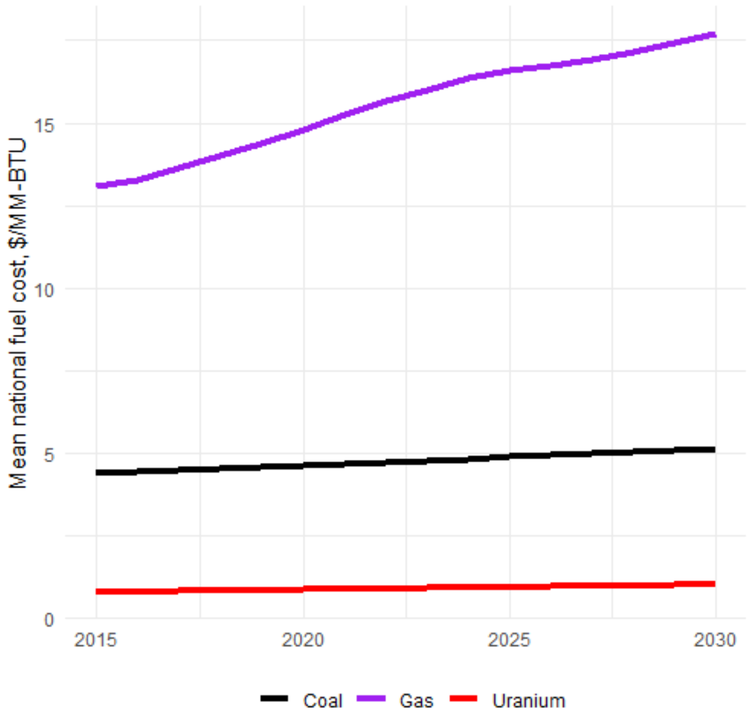

(b) Fuel costs

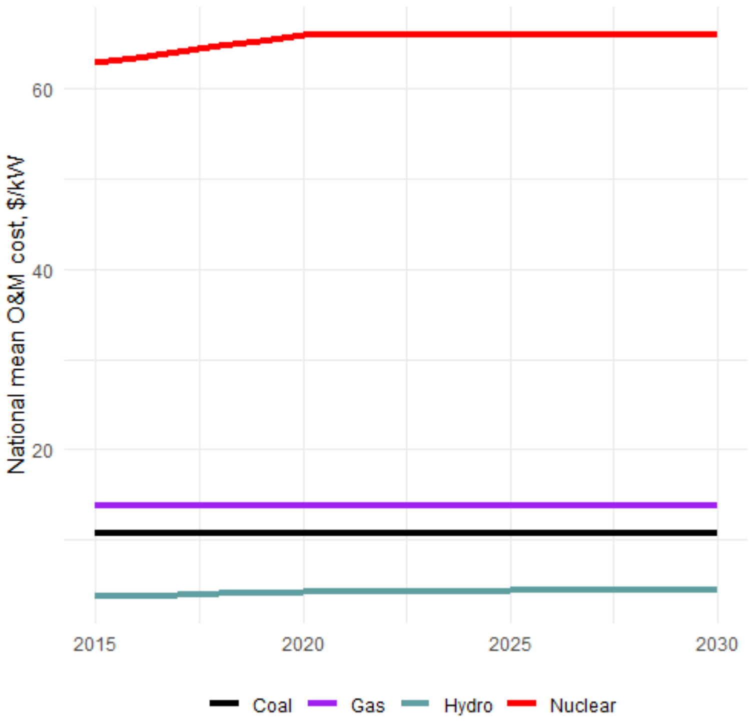

(c) Operation and maintenance costs

Supplementary Fig.1 Costs of various non-renewable technologies under all scenarios, 2015 to 2030.

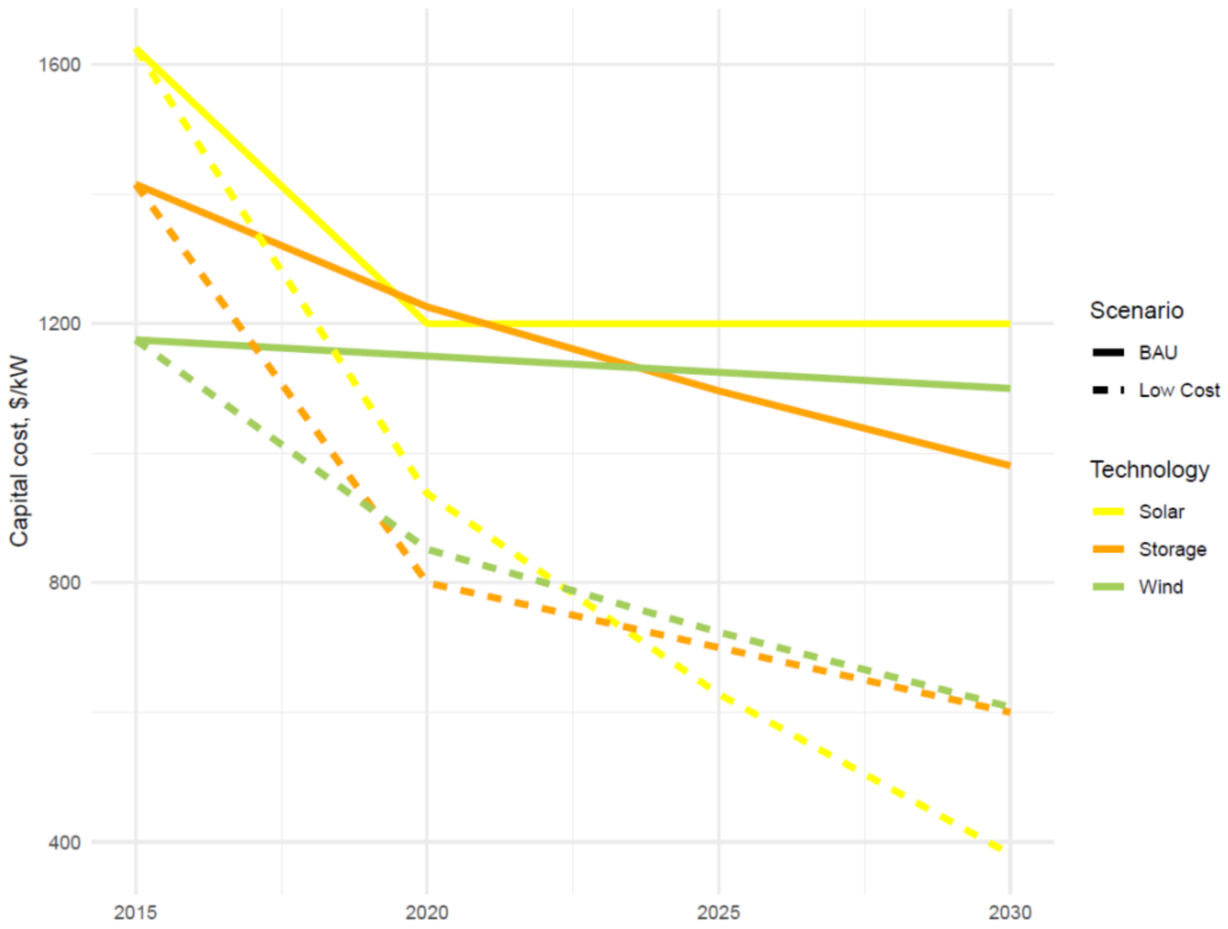

Supplementary Fig. 2 Capital costs of wind, solar, and storage technologies under BAU and Low Cost scenarios (R), 2015 to 2030. Capital costs of wind, solar, and storage technologies in the Carbon constraint scenario (C50) and Carbon constraint scenario (C80) are the same as in the Low Cost scenario (R).

Supplementary Tables

Supplementary Table 1 Summary of updates to the SWITCH-China model

Parameters	He et al., 2016	This study
Base year	2010	2015
Power plants	Plants level data up to 2010	Plants level data up to 2015, and updated future targets of hydro power, nuclear power, onshore wind, and solar PV, and storage
Transmissions	Transmission network up to 2010	Updated with new transmission plans, including new ultra-high voltage lines

Supplementary Notes

Supplementary Note 1: SWITCH-China model

He et al. first introduced the SWITCH-China model and studied the decarbonization of Chinese power sector.[1] We updated the SWITCH-China model in this paper. The objective function of the SWITCH-China model is to minimize the sum of (1) capital costs of existing and new power plants and storage projects; (2) fixed O&M costs incurred by all active power plants and storage projects; (3) variable costs incurred by each plant, including variable O&M costs, fuel costs to produce electricity and provide spinning reserves, and any carbon costs of greenhouse gas emissions; (4) capital costs of new and existing transmission lines and distribution infrastructure; and (5) annual O&M costs for new and existing transmission lines and distribution infrastructure. The below table presents the SWITCH-China calculations of the factors presented above.

$\min \boldsymbol{C}$	**Total cost**
$= \sum_{T,i} G_{T,i} \times c_{T,i}$	Generation capital costs
$+ \sum_{g,i} G_{g,i} \times x_{T,i}$	Generation fixed costs
$+ \sum_{T,i} O_{T,t} \cdot (m_{T,t} + f_{T,t} + C_{T,t}) \cdot hs_t$	Generation variable costs
$+ \sum_{a,a',i} T_{a,a',i} \cdot l_{a,a'} \cdot t_{a,a',i}$	Transmission and distribution costs

Where: T denotes generation technology, g denotes projects, i denotes time period, t denotes hourly time points, and a denotes load areas.

The model includes five primary sets of constraints: those that ensure that the load is satisfied; those that maintain the stipulated capacity reserve margin; those that require

maintaining operating reserves; those that enforce technology-specific targets (for example, wind and solar development plans, nuclear development plans, non-fossil energy targets, and other technology targets); and those that impose a carbon cap.

The SWITCH-China model employs multiple levels of temporal resolution to simulate power system dynamics throughout the period 2015 to 2030. The model considers investment periods in months, days, and hours. A single investment period contains historical data from 12 months, two days per month (the peak and median load days), and six hours per day. Each optimization considers three five-year investment periods: 2015 to 2020, 2020 to 2025, and 2025 to 2030, resulting in (3 investment periods) (12 months/investment period) (2 days/month) (6 hours/day) = 432 study hours during which the system is dispatched. Compared with simulating consecutive hours, simulating representative hours reduces computing time by a factor of 10, from 20 to 30 hours to about 2 to 3 hours. Additional study hours can be incorporated if the power system derived from the initial 432-timepoint optimization fails to meet load in any hour during the post-optimization dispatch check.

The output of generators that use renewable resources can be correlated not only among the sites of those resources but also with electricity demand. To account for those correlations, SWITCH-China employs time-synchronized historical hourly load and generation profiles for locations throughout China. Each date in a future investment period corresponds to an actual date from 2015 for which historical data are available regarding hourly loads, simulated hourly wind and solar capacity factors, and monthly hydroelectric availability. Hourly load data are scaled up to project future demand, while the availabilities of solar, wind, and hydroelectric resources are derived from historical data.

Supplementary Note 2: Updates to SWITCH-China model

Power plants

We add all coal power plants built from 2011 to 2015 as included in the Global Power Plant Database.[2] The provincial wind and solar capacity are updated from the National Energy Administration's 2015 wind and solar industry development brief and statistics, respectively. [3,4] The national planned solar, wind, hydro, and nuclear capacity are extracted from the Energy Production and Consumption Revolution Strategy (2016-2030) released by National Development and Reform Commission.[5] The technical parameters of power plants are assumed the same as in the He et al. paper.[1]

Transmissions

We updated the transmission lines, and new high-voltage direct current (HVDC) and high-voltage alternating current (HVAC) interprovincial lines are obtained from the corporate social responsibility reports of the State Grid, and China Southern Power Grid.[6,7] The capacities of inter-provincial transmission lines are between 4000MW-7500MW, rated at 500kV.[8] The trans-regional transmission capacities of the State Grid and China Southern Power Grid are 190 GW and 39.5 GW, respectively.[9] The costs of transmission are updated with the *Grid Project Construction Cost Analysis in the 12th Five-year Period* released by the China Electric Power Planning and Engineering Institute and China Renewable Energy Engineering Institute.[10]

Supplementary Note 3: Cost assumptions

Non-renewables

We first show the capital, operation and maintenance, and fuel costs associated with coal, gas, hydro and nuclear power plants in our model. We show the costs for solar, storage and wind, separately.

Capital costs

Capital costs are amortized over the expected lifetime of each generator or transmission line. Only those payments that occur during the period covered by the study are included in the SWITCH-China objective function.

Modeled capital costs for coal, gas, hydro and nuclear plants include trends to 2030 for different sizes and technologies of these plants. Supplementary Fig 1 shows the capital cost trend for the largest, and most common type of plant for each coal, gas, hydro, and nuclear power plant. Costs are assumed to increase for hydro and nuclear power plants but stay relatively constant for coal and gas plants between 2015 and 2030, respectively.

Fuel costs

Average national fuel costs for coal and gas in 2017 used in the SWITCH-China model are \$4.5/MMBtu and \$12.9/MMBtu, respectively. Fuel costs for coal, gas, and nuclear power plants all increase from 2017 to 2030 by 12.5, 23.7, and 21.4%, respectively. Provincial costs of coal are based on the national benchmark price at Qinhuangdao, minus/plus coal transportation costs. In 2030, coal, gas, and nuclear fuel costs increase to \$5.14, \$16.9, and \$0.98 per MMBtu, respectively.

Operation and maintenance costs

SWITCH-China uses operation and maintenance costs in addition to capital and fuel costs to calculate total system costs over a period of time. O&M costs are assumed to stay fairly

constant for coal, gas, and hydro power plants. Only nuclear power plants O&M costs see a slight increase between 2015 and 2030. Hydropower plants have the lowest O&M costs in 2030 with $4.5/kW. Coal operation and maintenance is slightly cheaper than gas-CC on a per kW basis, while nuclear is the most expensive unit to operate at $66/kW in 2030.

Renewables and Batteries

We propose two different cost trajectories for battery storage, solar, and wind power technologies. Under the BAU scenarios, costs fall but remain relatively high until pass 2030. The R, C50, and C80 scenarios assume that lower costs for storage, solar, and wind power technologies are expected.

Capital and O&M

Under the different scenarios the capital costs for solar, storage and wind technologies all decrease. Under the BAU scenario, we assume that capital costs in 2030 are lower than in 2015 by 26, 31, and 6% for solar, storage, and wind technologies, respectively. On the other hand, under the low-cost assumption, applied in the R and C scenarios in the main study, 2030 capital costs for solar, storage and wind, are lower than 2015 costs by 80, 57, and 66%, respectively.

Technology adoption, learning-by-doing, economies of scale, and manufacturing localization are driving the cost decease of wind technology[11], and similar effect could be found in the innovation and cost decease of solar PV[12], and storage[13]. Our capital costs assumptions for the Low Cost Renewable scenario for solar are a function of our estimates for the LCOE in 2030 expected given historical trends[14] and comparable with multiple renewable futures study.[15–17] The onshore wind and battery storage capital costs are informed by the 2018 NREL Annual

Technology Baseline study.[18] Supplementary Fig. 2 shows the capital costs assumptions of renewables and storage.

Our estimates for the O&M costs for these three technologies in our model are equal to 1% of the capital costs of that given technology for that given scenario.

Supplementary Note 4: CO_2 accounting

The paper uses the fuel emission factors as provided in the *China Provincial GHG Emission Inventory Guideline (in Chinese)* released by China's National Development and Reform Commission (NDRC) for emission calculation as posted below:

Coal: 25.41 kgC/GJ (93.17 kgCO_2/GJ)

Natural Gas: 15.32 kgC/GJ (56.22 kgCO_2/GJ)

Oil: 21.10 kgC/GJ (77.37 kgCO_2/GJ)

The total emissions are calculated with sum of plant level emissions from generation and spinning reserve provision and cannot exceed a pre-specified emission cap if a carbon constraint is introduced. [1]

We are aware of the importance of the life-cycle assessment (LCA) emissions of different technologies. The mean value of LCA emissions of solar and wind technologies are reported at 34.1gCO_2-eq/kWh and 49.9gCO_2-eq/kWh for wind and solar, respectively, according to a meta review.[19] In this study, we focus on the direct emissions so to make it more comparable to China's existing carbon mitigation goals. China's Intended Nationally Determined Contributions (INDCs) and existing carbon mitigation goals have not incorporated life-cycle carbon emissions. Future

studies are needed to address the question on how LCA emissions would impact power capacity expansion.

Supplementary Note 5: Demand assumptions

We project electricity consumption by province in 2030 using a log-linear regression model.[21,22] This model considers electricity consumption as a function of provincial gross domestic product (GDP), population, the percentage of total value added by tertiary industry out of total provincial GDP (tertiary share), and crude steel production. We assume that the average annual growth rate of GDP in each province from 2016 to 2020 follows the goal described in China's 13th Five-year Plan (FYP) for that province. We then assume that the average annual growth rate from 2021 to 2030 is half of that from 2016 to 2020. For provincial population, first we project population in 2020 based on each province's 13th FYP and then assume that from 2021 to 2030 population grows at half the rate assumed for 2016 to 2020. We fix the crude steel production in each province at its 2016 level. The log-linear model estimates a total of 8,757 TWh electrical usage in 2030 in China, which is close to LBNL's China 2050 Demand Resource Energy Analysis Model’s current policy scenario (8,595 TWh)[23] and to the State Grid Energy Research Institute model’s scenario 3, medium social-economic development (8,790 TWh). [24]

Supplementary References:

1. He, G. *et al.* SWITCH-China: A Systems Approach to Decarbonizing China's Power System. *Environ. Sci. Technol.* **50**, 5467–5473 (2016).
2. GEO, Google, KTH, Enipedia & WRI. *Global Power Plant Database*. http://datasets.wri.org/dataset/globalpowerplantdatabase (2018).
3. NEA. 2015 wind industry development brief. http://www.nea.gov.cn/2016-02/02/c_135066586.htm (2016).
4. National Energy Administration. Statistical Data of PV Generation in 2015 (in Chinese). http://sdb.nea.gov.cn/new/2016-2/20162594842.htm (2016).
5. NDRC. *Energy Production and Consumption Revolution Strategy (2016-2030)*. http://www.gov.cn/xinwen/2017-04/25/5230568/files/286514af354e41578c57ca38d5c4935b.pdf (2017).
6. China Southern Power Grid. *2018 Corporate Social Responsibility Report*. http://eng.csg.cn/Social_Responsibility/Social_Responsibility_Report/201905/P020190523361009432864.pdf (2019).
7. State Grid. *2017 Corporate Social Responsibility Report*. http://www.sgcc.com.cn/html/files/2018-08/29/20180829165036376453337.pdf (2018).
8. Geng, Q., Lu, Y., Fan, H. & Zhang, P. Comparative analysis of the system operation losses between UHV AC and EHV AC transmission systems. *Power System Protection and Control* 72–77 (2016).
9. CEC. *China Electricity Industry Annual Development Report 2019 (in Chinese)*. http://www.cec.org.cn/yaowenkuaidi/2019-06-14/191782.html (2019).

10. EPPEI & CREEI. *Grid Project Construction Cost Analysis in the 12th Five-year Period (in Chinese)*. (2017).

11. Nugent, D. & Sovacool, B. K. Assessing the lifecycle greenhouse gas emissions from solar PV and wind energy: A critical meta-survey. *Energy Policy* **65**, 229–244 (2014).

12. NDRC. *Enhanced actions on climate change: China's intended nationally determined contributions (in Chinese)*. http://www4.unfccc.int/submissions/INDC/Published%20Documents/China/1/China's%20INDC%20-%20on%2030%20June%202015.pdf (2015).

13. Qiu, Y. & Anadon, L. D. The price of wind power in China during its expansion: Technology adoption, learning-by-doing, economies of scale, and manufacturing localization. *Energy Economics* **34**, 772–785 (2012).

14. Zheng, C. & Kammen, D. M. An innovation-focused roadmap for a sustainable global photovoltaic industry. *Energy Policy* **67**, 159–169 (2014).

15. Kittner, N., Lill, F. & Kammen, D. M. Energy storage deployment and innovation for the clean energy transition. *Nature Energy* **2**, nenergy2017125 (2017).

16. Amol A. Phadke, Nikit Abhyankar, Ranjit Deshmukh, Julia Szinai & Anand R. Gopal. *Cost-effective decarbonization of California's power sector by 2030 with the aid of battery storage*. https://ies.lbl.gov/publications/cost-effective-decarbonization (2020).

17. NREL. *Renewable Electricity Futures Study*. https://www.nrel.gov/analysis/re-futures.html (2012).

18. Mai, T., Mulcahy, D., Hand, M. M. & Baldwin, S. F. Envisioning a renewable electricity future for the United States. *Energy* **65**, 374–386 (2014).

19. Williams, J. H. *et al.* The Technology Path to Deep Greenhouse Gas Emissions Cuts by 2050: The Pivotal Role of Electricity. *Science* **335**, 53–59 (2012).

20. NREL. *2018 Annual Technology Baseline*. https://data.nrel.gov/files/89/2018-ATB-data-interim-geo.xlsm (2018).

21. Lin, J., He, G. & Yuan, A. Economic rebalancing and electricity demand in China. *The Electricity Journal* **29**, 48–54 (2016).

22. Lin, J., Liu, X. & He, G. Regional electricity demand and economic transition in China. *Utilities Policy* **64**, 101047 (2020).

23. Zhou, N. *et al.* China's energy and emissions outlook to 2050: Perspectives from bottom-up energy end-use model. *Energy Policy* **53**, 51–62 (2013).

24. Hu, Z., Tan, X. & Xu, Z. *2050 China Economic Development and Electricity Demand Study (in Chinese)*. (China Electric Power Press, 2011).
